# Flavonoid Extract from Propolis Inhibits Cardiac Fibrosis Triggered by Myocardial Infarction through Upregulation of SIRT1

**DOI:** 10.1155/2018/4957573

**Published:** 2018-06-27

**Authors:** Qian Wang, Xin Sui, Dian-Jun Sui, Ping Yang

**Affiliations:** ^1^Department of Cardiology, China-Japan Union Hospital, Jilin University, Changchun 130033, China; ^2^Jilin Provincial People's Hospital, Changchun 130021, China; ^3^Baohua Hospital of Nongan County, Nongan 130200, China; ^4^College of Pharmacy, Changchun University of Chinese Medicine, Changchun 130021, China

## Abstract

The flavonoid extract from propolis (FP) has been shown to protect against heart injury induced by isoproterenol. However, the effect of FP on cardiac fibrosis after myocardial infarction (MI) as well as the underlying mechanisms is not known. In the present study, we used biochemical and histological approaches to examine the effects of FP on MI-induced cardiac fibrosis and the related mechanisms in a rat MI model and in angiotensin II- (Ang II-) treated rat cardiac fibroblasts (CFs).* In vivo,* MI was generated by ligation of the left anterior descending coronary artery of rats, which remained for 4 weeks. Rats were randomly divided into the sham, MI, FP (12.5 mg/kg/d), and MI+FP groups. We found that FP treatment improved heart function, reduced cardiac fibrosis, and downregulated the expression of fibrosis-related factors including collagen I, collagen III, matrix metalloproteinase-2 (MMP-2), MMP-9, transforming growth factor-*β*1 (TGF-*β*1), and p-Smad2/3, which coincided with the upregulated expression of silent information regulator 1 (SIRT1) in the hearts of MI rats. Our* in vitro* experiments showed that FP inhibited the proliferation and migration of primary cultured rat CFs and downregulated the expression of the above-mentioned fibrosis-related factors in Ang II-stimulated CFs. In addition, FP can decrease ROS production induced by MI and Ang II* in vivo* and* vitro*. Notably, silencing SIRT1 counteracted the FP-induced effects on CFs treated with Ang II. We conclude that FP inhibits MI-induced cardiac fibrosis through SIRT1 activation and that FP represents a potential promising drug for the treatment of MI patients in the clinic.

## 1. Introduction

Myocardial infarction (MI) is the major cause of heart failure, which has a high morbidity and mortality [1, 2]. During the progression of heart failure induced by MI, cardiac fibroblasts (CFs) are activated, proliferate, and secrete proteins such as collagens; collagens constitute the extracellular matrix (ECM), which replaces necrotic cardiomyocytes as a means to maintain the myocardial structure [3]. However, hyperactivation of CFs results in excessive ECM protein accumulation and induces cardiac fibrosis [4], subsequently leading to impairment of myocardium elasticity and heart failure [5]. Also, excessive ECM accumulation can predispose the heart to arrhythmias [5]. Therefore, searching for effective drugs to delay and/or reduce myocardial fibrosis is an important and active area of research.

Flavonoids are polyphenolic phytochemicals that are widely distributed in vegetables and fruits [6, 7]. Previous studies have shown that flavonoids purified from different sources possess a wide range of biological activities, including antiarteriosclerosis, antioxidative stress, antiproliferation, anti-inflammation, and antidiabetes [8–12]. Recently, our research group successfully extracted flavonoids from propolis (FP), a bee-derived substance [13], and identified the major components in FP, including kaempferol, pinobanksin-3-*O*-acetate, galangin, pinocembrin, 12-acetoxyviscidone, isosakuranetin, and chrysin [14]. We also demonstrated that FP treatment protected the heart against isoproterenol-induced pathological cardiac hypertrophy in mice [14]. However, whether and how FP affects myocardial fibrosis induced by MI remain unknown.

Silent information regulator 1 (SIRT1) belongs to the mammalian sirtuin protein family and exhibits a wide variety of cellular activities [15]. Accumulating evidence suggests that SIRT1 is involved in the initiation and progression of a number of diseases, particularly fibrotic diseases such as liver fibrosis, cardiac fibrosis, and renal fibrosis [16–18]. For instance, Jie et al. have reported that upregulated SIRT1 expression inhibits the formation and development of cardiac fibrosis in a rat MI model [19]. However, whether SIRT1 can mediate the FP-linked cardiac protection both* in vitro* and* in vivo* has not been investigated.

In the present study, we aimed to determine whether FP exhibits antifibrotic properties in a rat MI model. We further investigated whether FP affects the production of collagens as well as the proliferation and migration of primary cultured CFs stimulated by angiotensin II (Ang II). Moreover, we explored whether SIRT1 is involved in FP-induced cardiac protection.

## 2. Materials and Methods

### 2.1. Preparation and Analysis of FP

We used the same FP as reported in our previous work and analysis of FP as previously described [14]. Propolis was obtained from the Changchun University of Chinese Medicine (number 1019907). Propolis powder was dissolved in 10x of 75% ethanol/water in order to extract the active fractions. After the solution was filtered and the filtrate was desiccated to become powder, 1 g of the powder was added to 100 mL chloroform-ethanol and 100 mL 1% NaOH solution. FP was obtained after another round of filtration and desiccation. After then, FP was dissolved in 0.5% carboxymethylcellulose sodium (CMC-Na), and 1 ml was used in animal experiments.

### 2.2. Generation of Rat Myocardial Infarction Model

Male Wistar rats (8 weeks old, 210–240 g) were purchased from the Experimental Animal Center of Jilin University. All animal experiments conformed to the Guide for the Care and Use of Laboratory Animals (National Research Council, Eighth Edition, 2011) and were approved by the Jilin University Ethics Committee. To generate the rat MI model, rats were anesthetized with an intraperitoneal administration of pentobarbital sodium (50 mg/kg), and the chest was opened at the fourth intercostal space, and the heart was exposed. A 6-0 polypropylene suture was used to ligate the left anterior descending (LAD) coronary artery at 2-3 mm from its origin. The rats in the sham-operated group underwent the same procedure except that no ligation was performed for the LAD, and they served as surgical controls. All rats were fed a standard diet and had access to food and water* ad libitum*. The MI rats were initially treated with vehicle or with different doses of FP (1, 2.5, 5, 12.5, 25, and 50 mg/kg/d, intragastrically, 7 days before inducing MI model) for 28 days. The appropriate dose (12.5 mg/kg/d) was finally selected based on the preliminary data showing the effective inhibitory effect of FP on the mRNA expression of collagen I/III and hydroxyproline content (see below). Finally, the following animal groups were used in this study: (1) sham-operated control group (sham group), (2) MI with vehicle group (MI group), (3) FP alone group (12.5 mg/kg/d), and (4) MI+FP group (12.5 mg/kg/d).

### 2.3. Echocardiography and Hemodynamic Measurements

Four weeks after MI surgery, rats were anesthetized with pentobarbital sodium as mentioned above. Cardiac function parameters, including left ventricular internal dimension in diastole (LVID(d)), left ventricular internal dimension in systole (LVID(s)), left ventricular ejection fraction (LVEF), and left ventricular fraction shortening (LVFS), were measured with a transthoracic echocardiography system equipped with a 15 MHz phased-array transducer (SONOS 5500, Hewlett-Packard, Andover, MA, USA).

In addition, a catheter was inserted into the left ventricle of the rat from the right carotid artery, through which the heart rate (HR), left ventricular end diastolic pressure (LVEDP), and cardiac contractility (dP/dt maximum and dP/dt minimum) were assayed and recorded with a PowerLab ML880 (AD Instrument, Australia).

### 2.4. Masson's Trichrome Staining

Rats were euthanized 4 weeks after the MI surgery, and the left ventricles were harvested and fixed in 4% paraformaldehyde, followed by paraffin embedding. Sections (5 *μ*m) were prepared, and Masson's trichrome staining was performed to assess the degree of cardiac fibrosis. The region of cardiac fibrosis was stained green, and the non-ECM myocardium was stained red. Images were acquired at 400× magnification and analyzed using Image-Pro Plus 6.0 software (Media Cybernetics, Rockville, MD, USA). The collagen fraction volume was calculated as the amount of fibrotic area stained green divided by the total area of the section.

### 2.5. CF Culture

CFs were obtained from 8-week-old male Wistar rats, as described previously [20]. Briefly, the left ventricles were harvested, cut into pieces, and digested with collagenase II. After centrifugation and resuspension, the adherent CFs were isolated. The CFs were incubated in Dulbecco's modified Eagle's medium (DMEM) containing 10% fetal bovine serum, and CFs with 2-3 passages were used in this study.

### 2.6. ROS and Malondialdehyde Assay

The contents of ROS and malondialdehyde (MDA) in myocardial tissue or CFs were measured with two commercial kits (Nanjing Jiancheng Bioengineering Institute, Nanjing, China). All operations are according to the manufacturer's instructions.

### 2.7. Proliferation Assay

The CFs were cultured in 96-well plates. After serum-starvation for 24 h, the CFs were treated with or without FP, followed by stimulation with or without Ang II for another 24 h. Next, Cell Counting Kit-8 solution was added to each well, according to the manufacturer's instructions (Dojindo, Kumamoto, Japan). The optical density was assayed at an absorbance of 450 nm with a microplate reader (Bio-Rad, Hercules, CA, USA).

### 2.8. Migration Assay

CF migration was assayed, as described previously [19]. The CFs were incubated in the upper Transwell chambers in serum-free DMEM. DMEM (containing serum ) with or without Ang II, Ang II plus FP, or FP was added to the lower Transwell chamber as a chemotactic stimulus, and the number of migrated CFs was counted at 8 h after treatment. The same method of detection was performed in SIRT1-siRNA transfection experiments.

### 2.9. Cell Cycle Analysis

Flow cytometry was used to analyze the cell cycle of CFs following different treatments. Briefly, after the aforementioned treatments, the CFs were collected, fixed in ice-cold 70% ethanol, and cultured overnight at 4°C. Then, the CFs were treated with RNase A and propidium iodide for 1 h at 37°C. Next, the CFs were treated with ethanol and stained with propidium iodide, and the cell cycle distribution was assayed using a Coulter Epics XL-MCL instrument (Becton Dickinson, Franklin Lakes, NJ, USA).

### 2.10. Quantitative RT-PCR (RT-qPCR)

RNA was isolated from the primary cultured cardiac fibroblasts using TRIzol reagent (Invitrogen, USA), according to the manufacturer's instructions. The RNA concentrations were measured using the optical density at 260/280 nm. RT-qPCR was performed using a SYBR® RT-PCR Kit (Takara, China) and an ABI Prism 7300 instrument (Applied Biosystems, USA). The primer sequences used in this study are listed in Table 1.

### 2.11. Western Blotting

Total protein was extracted from the noninfarct region of the left ventricle (LV) or the primary cultured CFs, and the protein concentration was measured by the bicinchoninic acid assay method. Equal amounts of protein were separated on 10% sodium dodecyl sulfate-polyacrylamide gels by electrophoresis and then transferred onto polyvinylidene difluoride (PVDF) membranes. Following blocking with 5% nonfat milk, the PVDF membranes were incubated with primary antibodies against SIRT1 (Cell Signaling Technology, USA), collagen I (Abcam, USA), collagen III (Proteintech Group, USA), transforming growth factor-*β*1 (TGF-*β*1), matrix metalloproteinase-2 (MMP-2), MMP-9 (all ABclonal, USA), Smad2/3, and p-Smad2/3 (all BioWorld, USA) at 4°C overnight. The membranes were washed, followed by incubation with the corresponding secondary antibodies for 2 h, and then the protein bands were visualized with chemiluminescence and quantified using ImageJ software (version 1.38x, NIH, USA).

### 2.12. Statistical Analysis

Data are presented as the mean ± standard error of the mean (SEM). Differences among experimental groups were tested by one-way analysis of variance followed by the post hoc Bonferroni test.* P* values < 0.05 were considered statistically significant. All analyses and graphs were completed using SPSS 17.0 software (SPSS Inc., Chicago, IL, USA) and Prism 6 software (GraphPad Software Inc., La Jolla, CA, USA).

## 3. Results

### 3.1. FP Attenuated the Expression of Collagen I and Collagen III and Hydroxyproline Content in Noninfarcted Myocardium

**Figure 1:**
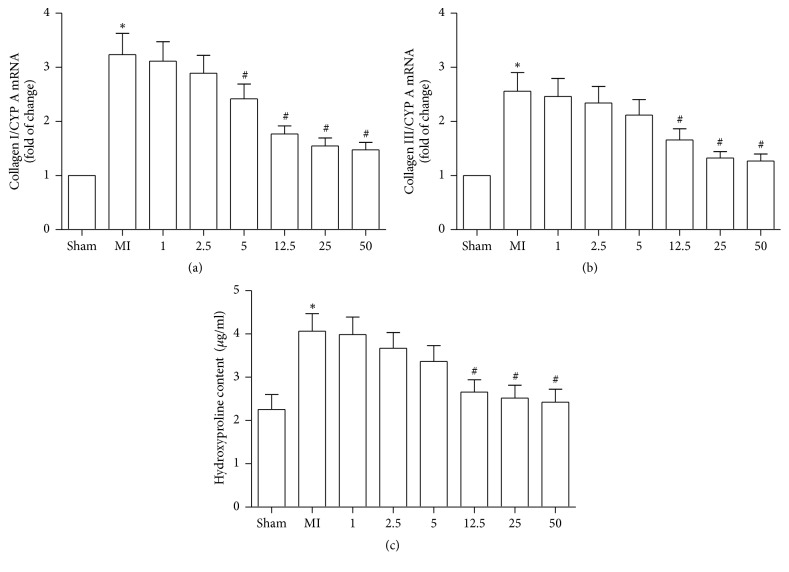
**FP decreased the expression of myocardial collagen I and collagen III and **hydroxyproline content** in MI rats.** Different concentrations of FP were tested to inhibit the expression of collagen I mRNA (a) or collagen III mRNA (b) and hydroxyproline content (c). Data are expressed as the mean ± SEM; *n* = 6 per group; ^*∗*^*P* < 0.05 versus the sham group; ^#^*P* < 0.05 versus the MI group.

First, we evaluated the effects of FP on the expression of collagen I/III mRNA and hydroxyproline (Hyp, a biomarker of total collagen content) content in the hearts of rats to determine the optimal dosage of FP which should be used in our study. Consistent with a previous report [21], the mRNA levels of collagen I/III and hydroxyproline content were significantly increased in the hearts of rats following MI, compared to the hearts of sham rats. FP treatment attenuated the MI-induced expression of collagen I/III mRNA and hydroxyproline content, with 12.5 mg/kg/d being the lowest dosage tested that efficiently inhibited the expression of collagen I/III mRNA and hydroxyproline content (Figure 1). No significant difference between FP, 12.5 mg/kg/d, and FP, 25 or 50 mg/kg/d, was observed (Figure 1). Based on these observations, we selected FP (12.5 mg/kg/d) as an appropriate concentration in the subsequent experiments.

### 3.2. FP Improved Cardiac Function of MI Rats

Four weeks after the operation, echocardiography revealed that LVEF and LVFS were significantly decreased, whereas LVID was significantly elevated in MI rats, compared with sham rats; but these changes were partially alleviated by FP treatment. No significant differences in these parameters were observed between the sham and FP alone groups. Similarly, hemodynamic examination showed that MI increased LVEDP and decreased dP/dt, and these changes were ameliorated by FP treatment (Table 2).

### 3.3. FP Attenuated Cardiac Fibrosis in MI Rats

**Figure 2:**
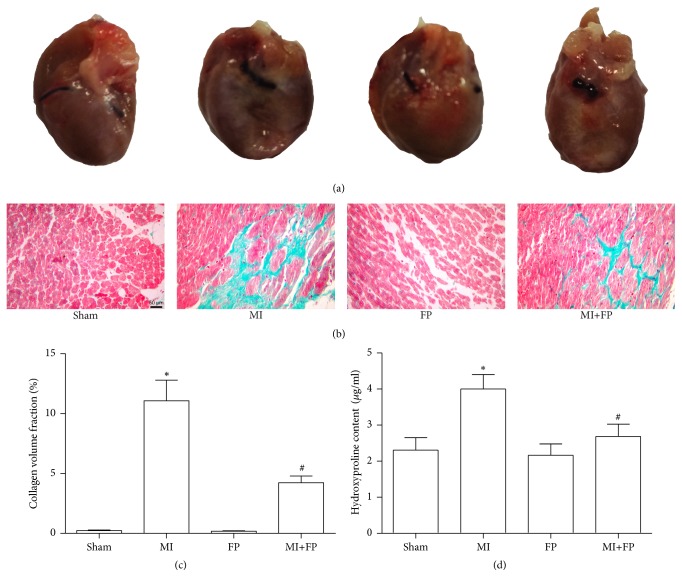
**FP reduced cardiac fibrosis in the LV noninfarct region after MI.** (a) Representative images showing morphological changes of rat hearts from the sham, MI, FP, and MI+FP groups. (b) Representative images showing cardiac fibrosis evaluated by Masson's trichrome staining. (c) Quantitative analysis of (b). (d) The hydroxyproline content was assayed in the LV of rats from the sham, MI, FP, and MI+FP groups. The results are expressed as the mean ± SEM; *n* = 6 per group; ^*∗*^*P* < 0.05 versus the sham group; ^#^*P* < 0.05 versus the MI group.

Next, we evaluated the effects of FP on cardiac fibrosis in the myocardium of MI rats. Compared to the sham rats, the area of cardiac fibrosis in the LV of MI rats was significantly increased as expected, but this increase was partially attenuated by FP treatment. There was no significant difference in the area of cardiac fibrosis and hydroxyproline content between the sham and FP alone groups (Figure 2).

### 3.4. FP Increased SIRT1 Expression and Decreased the Expression of MMP-2/9, TGF-*β*1, and p-Smad2/3 in MI Rats

**Figure 3:**
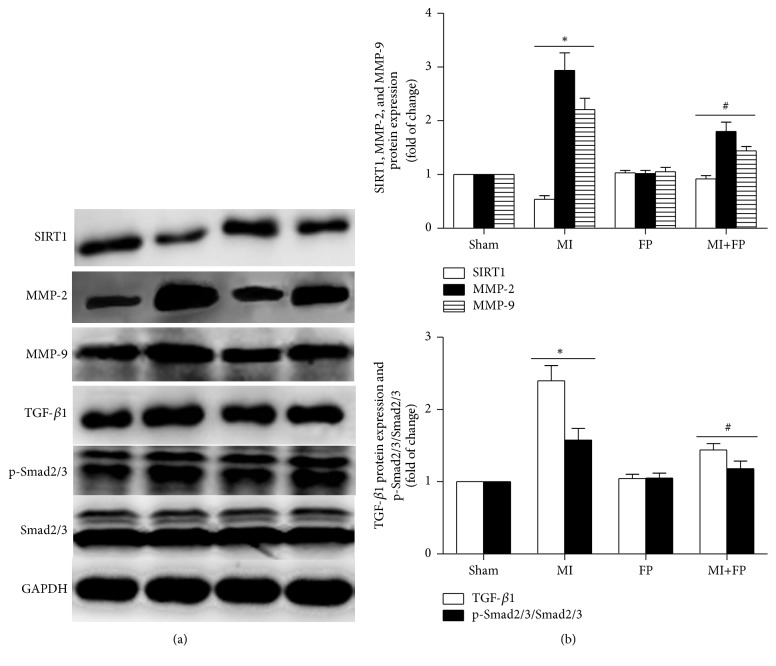
**FP increased SIRT1 expression but reduced the expression of **MMP-2/9, TGF-*β*1, and p-Smad2/3** in the hearts of MI rats.** (a) Representative Western blot images showing the effects of FP on the expression of SIRT1, MMP-2/9, TGF-*β*1, p-Smad2/3, and Smad2/3. GAPDH was used as an internal control. (b) Quantitative analysis of (a). Data are expressed as the mean ± SEM; *n* = 6 per group; ^*∗*^*P* < 0.05 versus the sham group; ^#^*P* < 0.05 versus the MI group.

To reveal the molecular basis underlying the protective effects of FP on the noninfarcted myocardium, Western blot analysis was used to analyze the changes in the expression of several factors that have been shown to be involved in MI-induced cardiac fibrosis. As shown in Figure 3, SIRT1 expression was significantly lower in the hearts of MI rats compared to that of sham rats. In contrast, the expression levels of cardiac fibrosis-related proteins such as MMP-2/9, TGF-*β*1, and p-Smad2/3 in the hearts of MI rats were higher than those in sham hearts. The above-mentioned abnormal changes were ameliorated with FP treatment, while no significant differences in the changes of these protein expression levels were observed between the sham and FP alone groups.

### 3.5. FP Increased SIRT1 Expression and Decreased the Expression of Collagen I/III, MMP-2/9, TGF-*β* 1, and p-Smad2/3 in Ang II-Stimulated CFs

**Figure 4:**
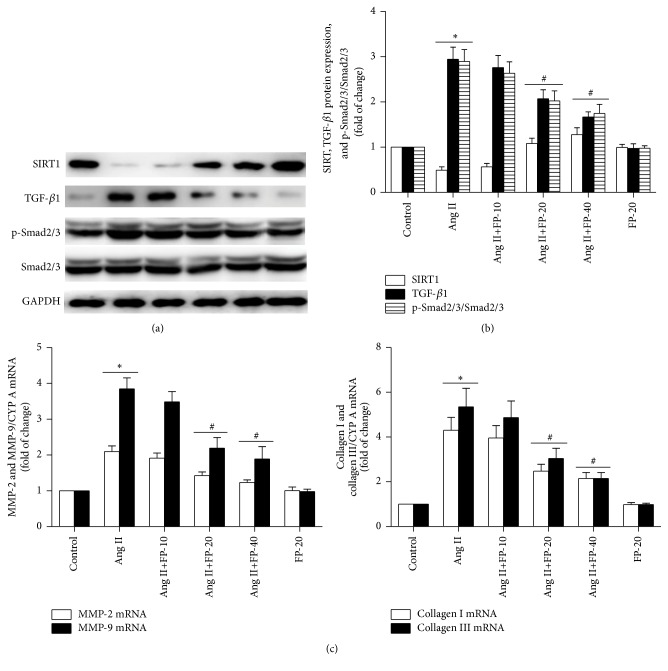
FP increased SIRT1 expression but reduced the expression of collagen I/III, MMP-2/9, TGF-**β**1, and p-Smad2/3 in Ang II-stimulated CFs. (a) Representative Western blot images showing different concentrations of FP on the expression of SIRT1, TGF-*β*1, p-Smad2/3, and Smad2/3. GAPDH was used as an internal control. (b) Quantitative analysis of (a). (c) RT-qPCR showing different concentrations of FP on the expression of collagen I/III and MMP-2/9. Data are expressed as the mean ± SEM; *n* = 6 per group; ^*∗*^*P* < 0.05 versus the control group; ^#^*P* < 0.05 versus the Ang II group.

In agreement with a previous report [19], Ang II stimulation significantly decreased SIRT1 expression in the primary cultured CFs compared to the vehicle group, while treatment with FP at 20 and 40 *μ*g/mL significantly attenuated the Ang II-induced reduction in SIRT1 expression in a dose-dependent manner. Also, Ang II stimulation increased the expression of these cardiac fibrosis-related factors, including collagen I, collagen III, MMP-2, MMP-9, TGF-*β*1, and p-Smad2/3, which was again attenuated by FP treatment (Figure 4).

### 3.6. SIRT1 Silencing Inhibited the Effects of FP on the Ang II-Induced Expression of Collagen I/III, MMP-2/9, TGF-*β*1, and p-Smad2/3

**Figure 5:**
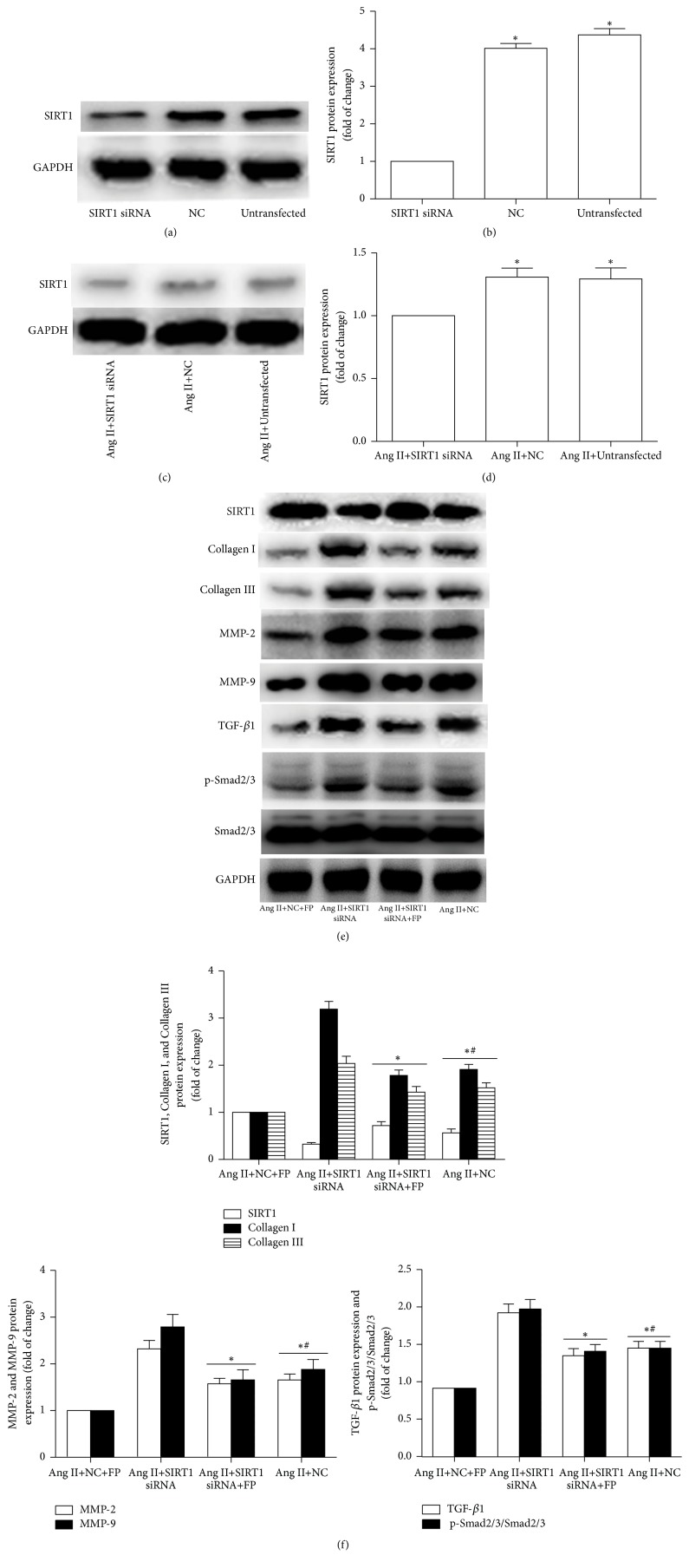
**SIRT1 knockdown suppressed FP-induced changes in the expression of SIRT1, collagen I/III, MMP-2/9, TGF-** **β** **1, and p-Smad2/3 in Ang II-treated CFs.** (a) The SIRT1 level was significantly knocked down by siRNA. (b) Quantitative analysis of (a). (c) The SIRT1 level was lower in the siRNA-transfected CFs compared to the NC and untransfected CFs stimulated by Ang II. (d) Quantitative analysis of (c). (e) Representative Western blot images showing that SIRT1-siRNA antagonized the FP-induced decrease in the expression of collagen I/III, MMP-2/9, TGF-*β*1, and p-Smad2/3. GAPDH was used as an internal control. (f) Quantitative analysis of (e). Data are expressed as the mean ± SEM; *n* = 6 per group; ^*∗*^*P* < 0.05 versus the SIRT1 siRNA, Ang II+SIRT1 siRNA, or Ang II+NC+FP group; ^#^*P* < 0.05 versus the Ang II+SIRT1 siRNA group. NC: the negative control group.

To explore whether SIRT1 is involved in mediating the FP-induced fibrosis inhibition, we transfected SIRT1-specific siRNA (SIRT-siRNA) or its negative control (NC) siRNA into cultured CFs. The protein level of SIRT1 was significantly lower in the SIRT1-siRNA group, compared to the NC and untransfected groups. In line with the above findings, FP treatment partially reduced the Ang II-activated expression of collagen I, collagen III, MMP-2, MMP-9, TGF-*β*1, and p-Smad2/3 in the NC group; but depletion of SIRT1 by siRNA suppressed this effect (Figure 5), thus implicating SIRT1 in the FP-triggered suppression of the expression of these factors induced by Ang II.

### 3.7. FP Inhibited Ang II-Induced CF Migration and Proliferation

**Figure 6:**
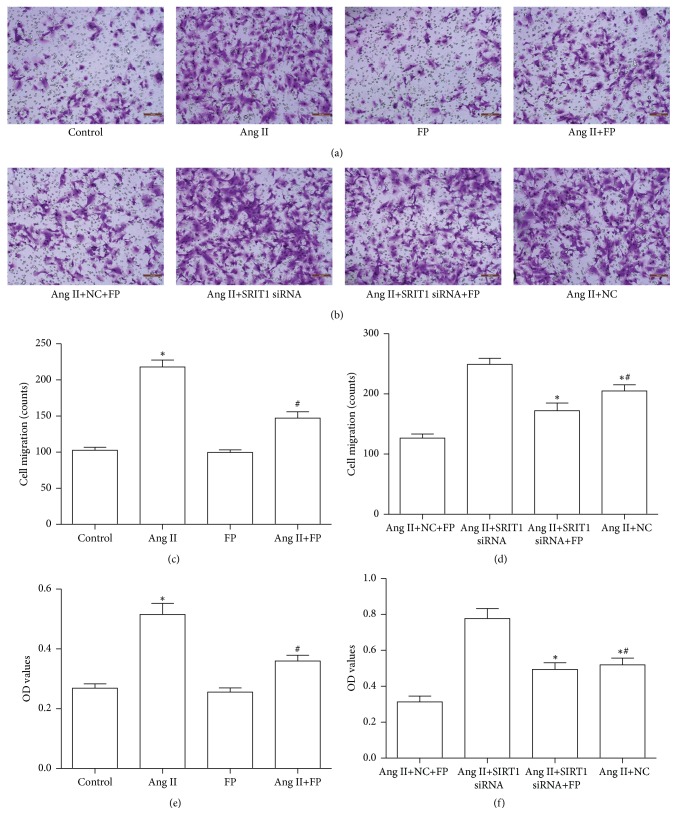
**FP suppressed the migration and proliferation of CFs induced by Ang II.** (a) Representative images showing that FP inhibited Ang II-stimulated CF migration. (b) Representative images showing that SIRT1-siRNA counteracted the effect of FP on CF migration. (c) Quantitative analysis of (a). (d) Quantitative analysis of (b). (e) FP inhibited Ang II-stimulated CF proliferation. (f) SIRT1-siRNA counteracted the effect of FP on CF proliferation. Data are expressed as the mean ± SEM; *n* = 6 per group; ^*∗*^*P* < 0.05 versus the control or Ang II+NC+FP group; ^#^*P* < 0.05 versus the Ang II or Ang II+SIRT1-siRNA group.

The maladaptive migration and overproliferation of CFs are essential for ECM remodeling following MI. We found that Ang II induced CF migration and proliferation, as reported previously [22], which was suppressed by FP treatment. However, knockdown of SIRT1 by siRNA suppressed the inhibitory effects of FP (Figure 6).

### 3.8. FP Arrested the Cell Cycle at the G0/G1 Phase in Ang II-Stimulated CFs

**Figure 7:**
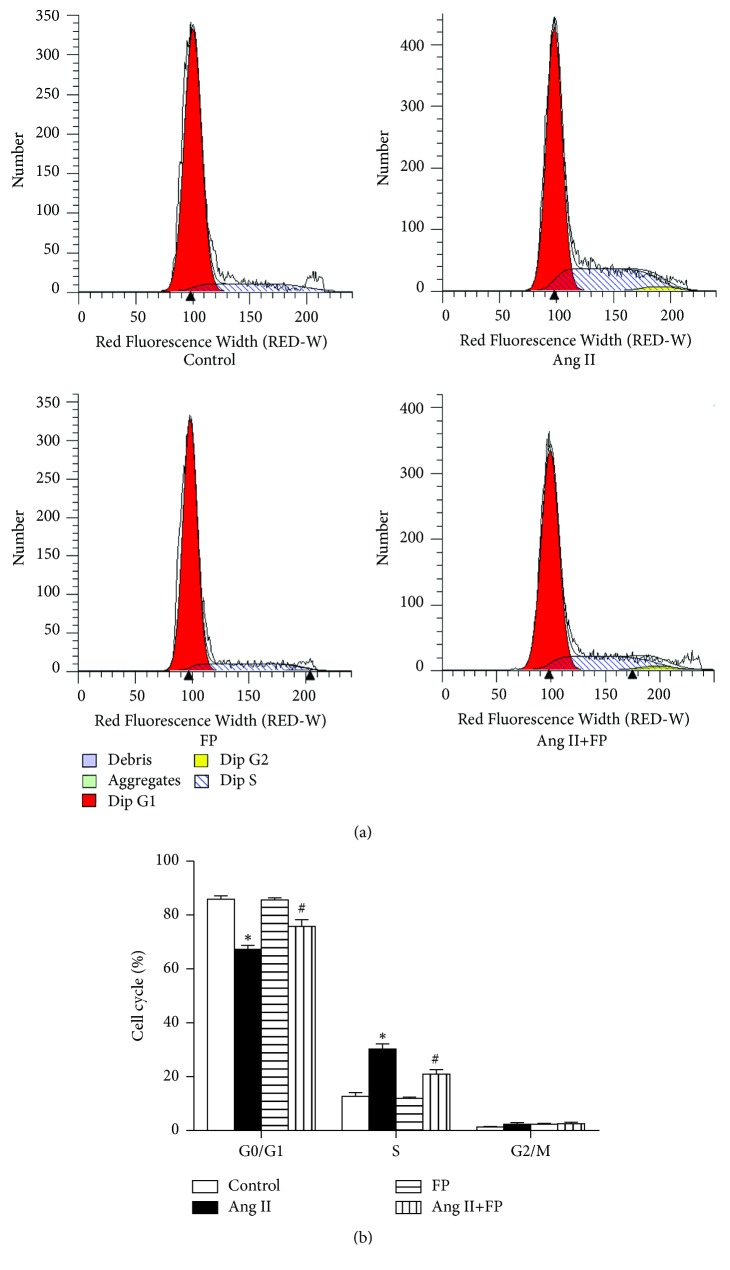
** FP suppressed the cell cycle progression of CFs induced by Ang II.** (a) Representative images showing the cell cycle distribution of the G0/G1, S, and G2/M phases. (b) Quantitation of the percentages of cell numbers in the G0/G1, S, and G2/M phases. Data are expressed as the mean ± SEM; *n* = 6 per group; ^*∗*^*P* < 0.05 versus the control group; ^#^*P* < 0.05 versus the Ang II group.

**Figure 8:**
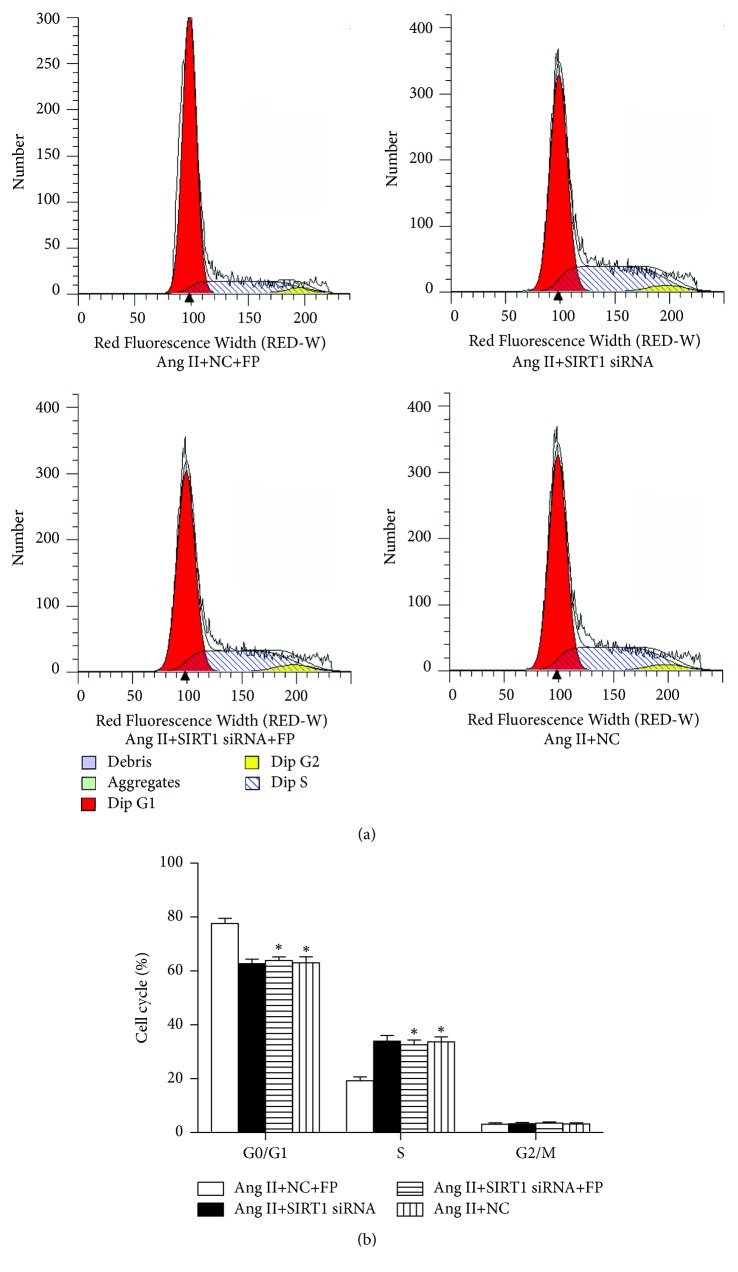
**SIRT1 knockdown inhibited the effect of FP on the cell cycle distribution of CFs induced by Ang II.** (a) Representative images showing the distribution of the G0/G1, S, and G2/M phases of each group. (b) Quantitation of the percentages of cell numbers in the G0/G1, S, and G2/M phases of each group. Data are expressed as the mean ± SEM; *n* = 6 per group. ^*∗*^*P* < 0.05 versus the Ang II+NC+FP group.

Next, we evaluated the effects of FP on CF cell cycle progression. Most CFs were in the G0/G1 phase in the vehicle-treated groups, while Ang II induced cell cycle progression from the G0/G1 phase to the S phase. The cell number in the S phase was significantly lower in the Ang II plus FP group, compared to the Ang II group (Figure 7). This inhibition imposed by FP treatment was abolished by SIRT-siRNA (Figure 8).

### 3.9. FP Decreased ROS and MDA Content in Myocardial Tissue of MI Rats and ROS Content in Ang II-Induced CFs

**Figure 9:**
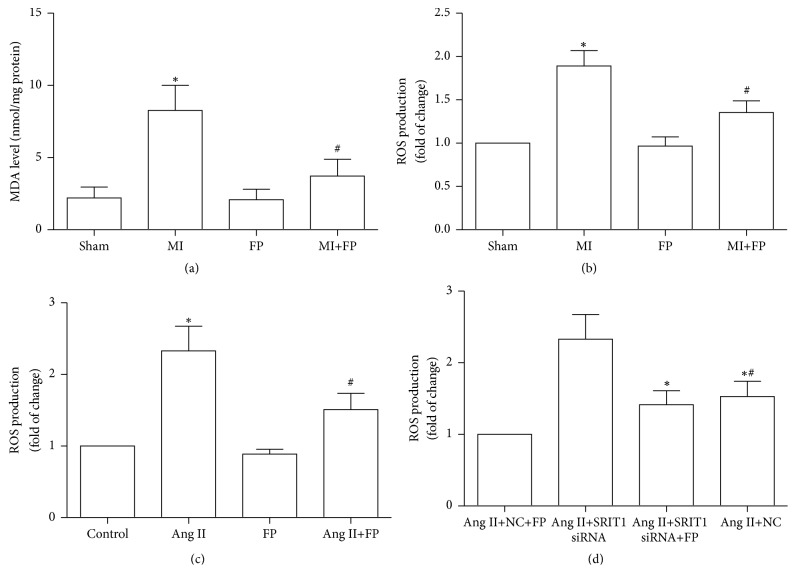
**FP decreased ROS and MDA content in myocardial tissue of MI rats and ROS content in Ang II-induced CFs.** (a) FP decreased MDA content in myocardial tissue of MI rats.(b) FP decreased ROS content in myocardial tissue of MI rats. (c) FP decreased ROS content in CFs induced by Ang II. (d) SIRT1-siRNA counteracted the effect of FP on ROS content in CFs induced by Ang II. Data are expressed as the mean ± SEM; *n* = 6 per group. ^*∗*^*P* < 0.05 versus the sham, control, or Ang II+NC+FP group; ^#^*P* < 0.05 versus the MI, Ang II, or Ang II+SIRT1-siRNA group.

ROS and MDA contents were markedly increased in myocardial tissue of MI rats, which was attenuated by FP treatment, while no significant differences in the changes of ROS and MDA contents in the sham and FP alone groups were observed. In addition, FP decreased ROS content in Ang II-induced CFs, and SIRT1 silencing partially inhibited the inhibitory effects of FP on ROS production (Figure 9).

## 4. Discussion

The major findings from the present study were as follows: (1) FP inhibited cardiac fibrosis and improved the cardiac function of MI rats; (2) mechanistically, FP elevated SIRT1 expression and downregulated the expression of fibrosis-related genes, including collagen I, collagen III, MMP-2, MMP-9, TGF-*β*1, and p-Smad2/3, in MI rats; (3) FP inhibited the proliferation and migration of CFs induced by Ang II; (4) FP elevated SIRT1 expression but inhibited the expression of the above-mentioned fibrosis-related factors in Ang II-stimulated CFs; (5) FP decreased ROS and MDA content in myocardial tissue of MI rats and ROS content in Ang II-induced CFs; and (6) knockdown of SIRT1 counteracted the effects of FP on the Ang II-activated CFs.

Cardiac fibrosis occurs when ECM synthesis exceeds degradation. Collagen I and collagen III are the main components of the ECM, and MMPs are the major enzymes in the ECM [23]. Previous studies have shown that both of them are increased in the heart after MI [24, 25] and that dysregulation of the expression of these proteins contributes to collagen remodeling and subsequent cardiac fibrosis following MI [26]. Propolis was accepted as a traditional medicine; proven to be beneficial to human health [27] and possess a wide range of biological and pharmacological activities, including antiatherosclerosis [28], antioxidative stress, and antihypertensive [29]. FP, the main bioactive compound of propolis [30, 31], has been shown to exhibit several cellular activities. For example, Maruyama et al. have demonstrated that FP plays a role in controlling the dilation of blood vessels, thereby decreasing blood pressure [32]; and research from our group has revealed that FP decreases the expression of collagen I and collagen III via the PI3K/AKT pathway in the hypertrophic ventricles of rats given isoproterenol [14]. The present study further showed that FP inhibited the changes in the myocardial expression of collagen I/III, MMP-2/9, TGF-*β*1, and p-Smad2/3 in MI rats. We further confirmed these observations in an* in vitro* model, in which the primary cultured rat CFs were pretreated with FP followed by Ang II stimulation. Ang II is well known to be involved in MI-induced cardiac remodeling [33, 34]. Therefore, FP ameliorates cardiac dysfunction following MI via reducing cardiac fibrosis.

Multiple cytokines including TGF-*β*1 are activated and participated in the development of cardiac fibrosis in MI rats. TGF-*β*1 contributes to CF proliferation and differentiation as well as the release of collagen to the ECM [35, 36]. Smad2/3 is one of the main downstream effectors of TGF-*β*1 [37]. Previous studies have reported that inhibition of TGF-*β*1/Smad2/3 signaling effectively protects against cardiomyocyte apoptosis and detrimental ventricular remodeling [37–39]. The* in vitro* experiment also suggested that the increased production of collagen I and collagen III as well as excessive proliferation of CFs induced by Ang II was attenuated through suppression of TGF-*β*/Smad signaling [40]. In the present study, we explored a functional linkage between FP and TGF-*β*1/Smad2/3 signaling both* in vitro* and* in vivo* and found that FP treatment downregulated TGF-*β*1 and p-Smad2/3 expression, indicating that FP inhibited cardiac fibrosis at least in part through repressing the activity of TGF-*β*1/Smad2/3 signaling.

SIRT1 belongs to the sirtuin protein family [41] and is well expressed in multiple tissues and organs including the heart, possesses a wide spectrum of biological activities, and is involved in the pathogenesis of cardiovascular diseases. For example, SIRT1 activation has been shown to improve heart function, reduce cardiac fibrosis, and inhibit TGF-*β*/Smad3 in doxorubicin-induced cardiomyopathy in rats [42]. Another study has reported that resveratrol can ameliorate mitochondrial dysfunction, decrease malondialdehyde and uncoupling protein 2 expression, increase peroxisome proliferator-activated receptor gamma coactivator 1-alpha deacetylation, and improve cardiac dysfunction and myocardial hypertrophy by upregulating SIRT1 expression in the myocardium of diabetic rats [43]. Also, recombinant human neuregulin-1*β* plays a beneficial effect on improving cardiac function and reversing cardiomyocyte hypertrophy and cardiac fibrosis, which is related to the activated ErbB2-ERK-SIRT1 signaling pathway in irradiation-induced heart damage [44]. In addition, L-arginine can decrease cardiac fibrosis as well as the expression of fibrosis markers, nuclear factor-*κ*B, and tumor necrosis factor-*α* by SIRT1 activation in STZ-induced diabetic rats [17]. Furthermore, Lai et al. have reported that inhibition of SIRT1/FoxO3a signaling pathway can decrease MnSOD transcription and exacerbate cell death and cardiac dysfunction in isoproterenol-induced cardiomyopathy rats [45]. In the present investigation, we observed that FP upregulated SIRT1 expression in the myocardium of MI rats and in the Ang II-stimulated CFs. We further confirmed the involvement of SIRT1 in the FP-linked inhibitory effects on Ang II-induced proliferation and migration of CFs by a loss of function study, showing that SIRT1 knockdown attenuated the FP-induced inhibitory effects. Hence, we provide direct evidence that SIRT1 mediates FP-induced cardiac protection against MI.

Ghazaal Moghaddam et al. reported that flavonoids played an antiproliferative role in malignant and normal cells [11]. Xiaofeng Shi et al. also demonstrated that the total flavonoids from the pine needles of* Cedrus deodara* inhibited the proliferation and retarded the HepG2 cells from G0/G1 phase to S phase. Since CFs proliferation was closely related to myocardial fibrosis after MI, we also examined the effect of FP on CFs proliferation. We found that FP possesses antiproliferative effect in Ang II-stimulated CFs through upregulation of SIRT1 expression [11].

Oxidative stress is an important mechanism in the development process of cardiac fibrosis after MI. Zeliha Selamoglu Talas et al. demonstrated that propolis treatment increased NO levels and decreased catalase and malondialdehyde levels in the heart tissue of L-NAME-induced oxidative injury rats [46]. Consistent with the above findings, we showed that FP exerted antioxidative stress effects through upregulation of SIRT1 expression.

In summary, we report here that FP inhibits cardiac fibrosis triggered by MI in rats. Mechanistically, FP induces SIRT1 expression and decreases the expression of collagen I/III, MMP-2/9, TGF-*β*1, and p-Smad2/3 as well as MDA and ROS content in the infarcted myocardium. Moreover, SIRT1 mediates the beneficial effects of FP. Thus, our study provides an experimental basis for FP to be used as a treatment for MI patients in the clinic.

## Figures and Tables

**Table 1:** Primer sequences used in the real-time quantitative PCR.

Target	Primers	Sequences
*Collagen I*	Forward	5′-TGGTCTTGGAGGAAACTTTGC-3′
	Reverse	5′-CTGTGTCCCTTCATTCCGG-3′
*Collagen III*	Forward	5′-ACCTGAAATTCTGCCACCCT-3′
	Reverse	5′-GCCTTGAATTCTCCCTCATTG-3′
MMP-2	Forward	5′-GGAAGCATCAAATCGGACTG-3′
	Reverse	5′-CACCCTCTTAAATCTGAAATCACC-3′
*MMP-9*	Forward	5′-AAGGATGGTCTACTGGCACA-3′
	Reverse	5′-TTGCGTTTCCAAAGTAAGTG-3′
CYP A	Forward	5′-TATCTGCACTGCCAAGACTGAGTG-3′5′-CTTCTTGCTGGTCTTGCCATTCC-3′
	Reverse	

**Table 2:** Echocardiographic and hemodynamic measurements.

	Sham	MI	FP	MI+FP
Echocardiography				
LVEF (%)	65 ± 2.13	34 ± 2.34_ _^*∗*^	67 ± 2.44	46 ± 2.28^#^
LVFS (%)	36 ± 1.98	19 ± 1.97_ _^*∗*^	37 ± 1.64	27 ± 2.11^#^
LVID(d) (mm)	6.82 ± 0.22	9.93 ± 0.29_ _^*∗*^	6.91 ± 0.35	8.55 ± 0.36^#^
LVID(s) (mm)	3.40 ± 0.20	8.22 ± 0.31_ _^*∗*^	3.41 ± 0.30	6.20 ± 0.26^#^
Hemodynamic				
HR (bpm)	397 ± 15	414 ± 14	399 ± 13	407 ± 17
dP/dt max (mm Hg/s)	5559 ± 153	3416 ± 174_ _^*∗*^	5527 ± 169	4513 ± 181^#^
dP/dt min (mm Hg/s)	3977 ±166	2435 ± 172_ _^*∗*^	3936 ± 171	3411 ± 183^#^
LVEDP (mmHg)	7.57 ± 0.29	14.12 ± 0.57_ _^*∗*^	7.84 ± 0.35	9.01 ± 0.28^#^

LVEF, left ventricular ejection fraction; LVFS, left ventricular fraction shortening; LVID(d), left ventricular internal dimension in diastole; LVID(s), left ventricular internal dimension in systole; HR, heart rate; dP/dt max, maximal slope of systolic pressure increment; dP/dt min, minimal slope of diastolic pressure decrement; LVEDP, left ventricular end-diastolic pressure. Data are means ± SEM. _ _^*∗*^ *P* < 0.05 versus the sham group; ^#^*P* < 0.05 versus the MI group.

## Data Availability

The data used to support the findings of this study are available in this article.
